# The Functions of Long Non-Coding RNA during Embryonic Cardiovascular Development and Its Potential for Diagnosis and Treatment of Congenital Heart Disease

**DOI:** 10.3390/jcdd6020021

**Published:** 2019-06-01

**Authors:** Nadia Turton, Ross Swan, Thanujan Mahenthiralingam, Dominic Pitts, Iain M. Dykes

**Affiliations:** Pharmacy and Biomolecular Sciences, Liverpool John Moores University, Liverpool L3 3AF, UK; N.Turton@2015.ljmu.ac.uk (N.T.); R.C.Swan@2016.ljmu.ac.uk (R.S.); T.Mahenthiralingam@2016.ljmu.ac.uk (T.M.); D.Pitts@2016.ljmu.ac.uk (D.P.)

**Keywords:** non-coding RNA, lncRNA, miRNA, PRC2, congenital heart disease, heart development, epigenetics, embryonic stem cell, patterning, gene regulation

## Abstract

Congenital heart disease (CHD) arises due to errors during the embryonic development of the heart, a highly regulated process involving an interplay between cell-intrinsic transcription factor expression and intercellular signalling mediated by morphogens. Emerging evidence indicates that expression of these protein-coding genes is controlled by a plethora of previously unappreciated non-coding RNAs operating in complex feedback-control circuits. In this review, we consider the contribution of long non-coding RNA (lncRNA) to embryonic cardiovascular development before discussing applications to CHD diagnostics and therapeutics. We discuss the process of lineage restriction during cardiovascular progenitor cell differentiation, as well as the subsequent patterning of the cardiogenic progenitor fields, taking as an example the regulation of NODAL signalling in left-right patterning of the heart. lncRNA are a highly versatile group. Nuclear lncRNA can target specific genomic sequences and recruit chromatin remodelling complexes. Some nuclear lncRNA are transcribed from enhancers and regulate chromatin looping. Cytoplasmic lncRNA act as endogenous competitors for micro RNA, as well as binding and sequestering signalling proteins. We discuss features of lncRNA that limit their study by conventional methodology and suggest solutions to these problems.

## 1. Introduction

Congenital heart disease (CHD) is the most common birth defect, occurring in 0.6–0.9% of live births [1,2]. Additionally, as many as 7.5% of new-borns may have milder undiagnosed forms of CHD, such as bicuspid aortic valve and minor ventricular septal defects, many of which may have no noticeable effect on health [1]. As a result of improved treatment, many patients with CHD now survive into adulthood and require lifelong care [2]. CHD arises from errors during embryonic development of the heart, and therefore a knowledge of gene regulation and patterning during development is central to an understanding of CHD aetiology and diagnosis. Similarly, emerging treatment options such as the grafting of stem cell derived engineered tissue—which offers the hope of a lifelong treatment because the grafted tissue can grow as the heart enlarges during adolescence [3]—requires a knowledge of the embryonic gene regulatory processes which direct the differentiation of pluripotent stem cells towards cardiovascular lineages.

Long non-coding RNA (lncRNA) are a large and diverse group of regulatory RNAs. Estimates of their prevalence vary, but some studies have suggested that much of the non-coding genome is transcribed at some point during development [4,5]. Beyond their lack of a protein-coding function and their being of a size larger than the better characterised micro RNA (miR)—lncRNA are defined as being greater than 200 nucleotides in length—there is little to unify this group. In broad terms, their regulatory functions may be divided into nuclear (or pre-transcriptional) functions and cytoplasmic (or post-transcriptional) roles (Table 1) [6]. In the nucleus, lncRNA are able to regulate transcription, often by acting as a scaffold to assemble large chromatin remodelling complexes. As we shall see, they may also be involved in targeting these complexes to specific sites in the genome. At the post-transcriptional level, many lncRNA are believed to bind to and thus negatively regulate miR, competing with messenger RNA (mRNA) for available miRs [6].

In this review, we will consider the process of lineage restriction in the early embryo which leads to the emergence of a cardiovascular progenitor cell. Over the last 25 years or so, observations of the development of normal embryos and those carrying targeted mutations, together with *in vitro* differentiation studies, have allowed us to build an understanding of the molecular mechanisms controlling this process. We now have a reasonably good understanding of how induction and patterning of progenitor fields by morphogens leads to the expression of transcription factors that drive differentiation. Recent evidence has demonstrated a previously unappreciated role for lncRNA in a permissive and feedback role. Exciting evidence suggests that lncRNA may regulate epigenetic patterning of the genome to control fate specification. We will then go on to explore the role of lncRNA in patterning of the emerging cardiogenic fields, taking as our example the process of left-right axis specification by the NODAL pathway, disruption of which is linked to many complex forms of CHD [7,8]. We will then consider some of the problems and challenges involved in lncRNA research, before finally going on to review studies which have explored the potential of lncRNA as a biomarker for liquid biopsies, as well as possible therapeutic approaches targeting lncRNA.

## 2. Early Heart Development: Embryonic Specification of Cardiovascular Lineages

With the exception of the cardiac neural crest, all cardiovascular lineages are derived from the mesoderm germ layer. The mesoderm in mammalian embryos is derived from a transient population of cells, the mesendoderm. The mesendoderm arises from pluripotent epiblast cells and gives rise to both mesoderm and endoderm as cells ingress through the primitive streak during gastrulation. The mesoderm is induced and simultaneously patterned along the anterior-posterior axis by gradients of morphogens (primarily of the Wingless / INT (WNT), Bone Morphogenetic Pathway (BMP) and Transforming Growth Factor (TGFβ) families) [9]. Cells located within the anterior primitive streak form paraxial mesoderm (which gives rise to the somitic musculature), while cells located in the mid or posterior primitive streak give rise to lateral plate mesoderm, from which the cardiogenic mesoderm is derived [10]. Lineage tracing suggests that the heart is derived from 250 cardiac precursors [11]. These precursors migrate laterally to form two distinct heart fields known as the primary and secondary fields. The earliest beating heart structure formed in the embryo is a linear heart tube, and this later undergoes a complex process of looping and remodelling to create the adult four-chambered morphology. The linear heart tube is formed by cells originating in the primary heart field, and these cells contribute primarily to the left ventricle of the mature heart. Cells from the second heart field migrate into the heart tube a day or so later and contribute to the future right ventricle and outflow tract.

Cardiogenic specification seems to be determined not by a single factor but by a network of cross-regulatory transcription factors, the cardiogenic gene regulatory network (CGRN), which has been strongly conserved throughout evolution [12,13]. NKX2.5 and GATA4 are common to the CGRNs operating in both heart fields, while TBX5 is part of the first heart field network and a number of factors, including MEF2C make up the second field CGRN. The network operating in the *Drosophila* heart is similar to that in the mammalian heart fields. Recent work aimed at identifying the minimum number of factors required to transdifferentiate fibroblasts into cardiomyocytes has shed light on the core of this CGRN. This has shown that the reprogramming of neonatal fibroblasts can be achieved using GATA4, TBX5 and MEF2C [14] (a combination not seen in nature, comprising factors from the first and second heart fields), while the reprogramming of adult cardiac fibroblasts required the addition of HAND2 [15].

Work in *Drosophila* has shown that Tinman (NKX2.5) acts a master regulator to initiate expression of the CGRN in flies and *Tinman* mutants, which as the name suggests, completely lack a heart [16]. This function is not conserved in vertebrates. The T-box transcription factor EOMES (Eomesodermin) is the first specific marker of the cardiogenic lineage in mammals. EOMES is expressed within the primitive streak at E5.75 and activates expression of the transcription factor MESP1 in cardiogenic precursors at the primitive streak stage [17]. MESP1 in turn activates the CGRN [18] as cardiac precursors migrate laterally to form the heart fields [19]. MESP1 expression is initiated at E6.5 in mice in a subset of mesoderm cells, including but not limited to the cardiogenic precursors; it is expressed as they ingress through the primitive streak but is then rapidly downregulated by E7.5 [19]. Although MESP1 has the properties of a master regulator of cardiac specification [18,20] it should be noted that its expression is not limited to cardiovascular progenitors [19] and, furthermore, that forced expression of MESP1 alone is insufficient to drive transdifferentiation of dermal fibroblasts [21]. Single cell sequencing has revealed unexpected diversity even in MESP1+ cardiogenic precursors, suggesting that regional differences in lineage specification occur early in cardiogenic precursors and that the segregation into primary and secondary heart field lineages may precede MESP1 expression [22,23].

## 3. The Role of lncRNA in Cardiogenic Lineage Specification

### 3.1. Roles in Specification of Cardiogenic Mesoderm

The lncRNA *Bvht* (Braveheart) is expressed in mouse pluripotent stem cells, before subsequently becoming restricted to the cardiogenic lineage during gastrulation [24]. Knockdown of *Bvht* using short hairpin RNA (shRNA) results in a failure to develop contractile tissue when stem cells are differentiated within embryoid body cultures [24]. *Bvht* appears to be required for *Mesp1* expression because knockdown embryoid bodies show loss of *Mesp1* expression as well as loss of downstream genes within the CGRN (e.g., *Nkx2.5, Gata4, Hand1/2, Tbx5*) and subsequently lack of markers of differentiated cardiomyocytes such as *Myh7* [24]. The phenotype can be rescued by forced expression of *Mesp1* [24].

*Bvht* appears to play a permissive rather than instructive role in cardiac specification because it is expressed in pluripotent cells and its expression is lost in non-cardiogenic lineages. Furthermore, unlike *Mesp1*, which is expressed only within a brief time window during cardiac specification [19], expression of *Bvht* persists into adulthood [24]. Paradoxically, *Bvht* appears to achieve this permissive role by mediating transcriptional repression. *Bvht* has been shown to interact both in embryonic stem cells and at the embryoid body stage with SUZ12, a component of the polycomb repressive complex 2 (PRC2) [24]. PRC2 adds di- or tri-methyl marks to lysine 27 of histone H3 in order to repress gene expression [25]. The ability to interact with chromatin remodelling complexes appears to be a mechanism of action common to many lncRNAs [26,27]—one study found that as many as 20% of known lncRNAs can interact with PRC2 [28].

Thus, recruitment of PRC2 by *Bvht* leads to deposition of the repressive histone mark H3K27Me3. How does *Bvht* mediate a permissive function through transcriptional repression? Klattenhoff et al. reconcile this apparent contradiction by suggesting that *Bvht* regulates bivalent histone marks at primed loci (loci which carry both repressive and activating histone marks, labelling them for transcriptional activation at a later stage) during differentiation. In support of this hypothesis, they demonstrate that in *Bvht* shRNA embryoid bodies, cardiogenic genes—including *Mesp1, Nkx2.5* and *Hand1/2*—remain in a bivalent state and associated with SUZ12 [24]. Thus, it appears that the presence of the lncRNA is required to remove PRC2 from cardiogenic loci, permitting progression towards the cardiogenic cell fate.

A follow-up study conducted by the same group has demonstrated that in addition to this pre-transcriptional function in gene regulation, *Bvht* has a second independent post-transcriptional role. They described a motif located at the 5′ end of *Bvht* which they named AGIL, or 5′ asymmetric G-rich internal loop [29]. This motif is able to bind to CNBP (CCHC-type zinc finger nucleic acid binding protein), a zinc finger protein which has been show to bind to mRNA, regulating mRNA stability and functioning in post-transcriptional gene regulation [30]. Cardiogenic differentiation of pluripotent cells carrying a CRISPR-targeted deletion of the AGIL motif within *Bvht*—but otherwise normal—revealed that while expression of *Mesp1* was unaffected by this loss, expression of the downstream CGRN was reduced [29]. In contrast, targeted mutation of *Cnbp* itself had the opposite effect and led to increased expression of the CGRN [29]. *In situ* hybridisation has shown that *Cnbp* is expressed briefly in the anterior (somitic) mesendoderm at E7.5, but there is no evidence for expression in the cardiogenic mid/posterior primitive streak or later heart fields, and by E8.25 expression is restricted to the headfolds and prospective neural tissue [31]. Thus *Cnbp* appears to act in non-cardiogenic lineages to suppress the CGRN. Loss of *Cnbp* partially rescues the phenotype of AGIL deleted cells [29], suggesting an antagonistic interaction between these two factors which may mediate the cardiogenic mesoderm/axial mesoderm choice point during gastrulation. A second lncRNA—*LAST* (lncRNA-assisted stabilization of transcripts)—has been shown to cooperatively bind with CNBP to *CCND1* (Cyclin D1) mRNA to promote mRNA stability during cell cycle control [32], suggesting this may be a general lncRNA mechanism.

**Figure 1:**
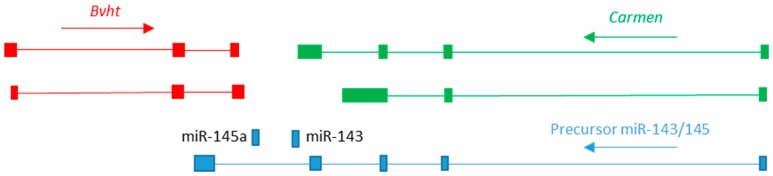
Organisation of the mouse *Braveheart/Carmen* locus. Cartoon to illustrate the genomic organization. Each long non-coding RNA (lncRNA) is shown in a different colour and arrows indicate direction of transcription.

The studies discussed above have begun to unravel how one lncRNA can regulate lineage restriction. However, several pieces of the puzzle are missing. The exact relationship between the pre- and post-transcriptional functions of *Bvht* is unclear. It is also unclear what triggers loss of *Bvht* expression in non-cardiogenic lineages and how a bivalent histone state is maintained in pluripotent cells despite expression of *Bvht*. Answers to some of these questions may come from an examination of the wider genomic context. *Bvht* is located in a complex genomic locus rich in lncRNA (Figure 1), and evidence is accumulating that its expression may be regulated by complex cis-regulatory interactions between these lncRNAs. Immediately distal to *Bvht* are the micro RNAs miR-145a and miR-143, which are derived from an intron of a precursor primary miRNA (pri-miR) lncRNA of otherwise-unknown function containing multiple exons and a polyadenylation signal [33]. *In vitro* differentiation studies show that miR-145a and miR-143 drive acquisition of the vascular smooth muscle cell (VSMC) fate [34]. Analysis of a mouse model in which the miR-145a/143 sequence within the pri-miR was replaced with a lacZ cassette revealed that VSMCs are produced, but that they remain in a proliferative, undifferentiated state [35]. Expression of the pri-miR appears to be driven by a large, conserved upstream enhancer region, and a transgenic reporter of this enhancer shows expression in the heart fields of the E7.5 cardiac crescent stage embryo and also widespread expression in the early heart tube, before later becoming restricted to the smooth muscle lineage [34]. This enhancer has subsequently been shown to have properties of a “super-enhancer”, that is to say, a cluster of linked enhancer regions [36]. Super-enhancers were first described in the regulation of pluripotency and are defined as being an order or magnitude larger than regular enhancers and in being bound by Mediator complex at a level also an order of magnitude greater [37].

Enhancer RNAs (eRNAs) are lncRNAs transcribed from active enhancers and are hypothesised to function in regulating conformational changes in chromatin required to bring the enhancer into apposition with its promoter [38,39]. A recent study demonstrated that a large number of cardiogenic enhancers are associated with eRNA expression [39]. The activity of the miR143/miR145a super-enhancer has been shown to be linked to expression of an eRNA named *Carmen* (cardiac mesoderm enhancer-associated noncoding RNA), a differentially spliced lncRNA overlapping the miR143/miR145a locus (Figure 1) [40]. The expression of *Carmen* in mouse embryonic carcinoma PC19CL6 cells differentiated towards a cardiomyocyte cell fate is correlated with activity of the enhancer as shown by H3K27Ac marking [40]. shRNA knockdown of *Carmen* in these cells also knocks down *Bvht* expression [40]. In addition, the expression of *Eomes*, a transcription factor transiently expressed in primitive streak and upstream of *Mesp1* [17], is also reduced, while expression of the pluripotency factors *Oct4* and *Nanog* is increased [40]. Knockdown of *CARMEN* in human cells does not affect miR143/145 expression [40], yet these are lost in *Bvht* shRNA embryoid bodies [24]. RNA immunoprecipitation indicated that, like *Bvht, Carmen* is also associated with the PRC2 complex [40]. Together, these data suggest a regulatory feedback mechanism is operating to control expression of these three lncRNAs during cardiogenic specification.

The binding of EOMES to the *Mesp1* promoter is also regulated by *Linc1405*, an intergenic lncRNA located between *Eomes* and *Cmc1* [41,42]. Like *Bvht*, *Linc1405* also has a permissive role in cardiogenic differentiation and knockdown results in loss of *Mesp1* expression [42]. EOMES binds to two conserved T-box core motifs within the *Mesp1* promoter [17]. H3K27Ac and H3K4Me are reduced at these sites in the *Linc1405* knockdown and EOMES binding is reduced [42]. The Trithorax group/MLL protein complexes (TrxG/MLL) are a diverse group of chromatin remodelling complexes which oppose the inhibitory action of PRC2, often by methylating lysine 4 of histone H3 [43]. Guo et al. show that *Linc1405* is responsible for assembling a transcriptional activation complex consisting of EOMES and the trithorax complex proteins WDR5 and GCN5 necessary for *Mesp1* activation in the primitive streak [42].

### 3.2. Regulation of the Mesoderm-Endoderm Choice Point

Mesendoderm is formed from the epiblast during gastrulation and gives rise to both mesoderm and endoderm. Mesendoderm is characterised by expression of genes such as *EOMES, GSC* (Goosecoid) and *T* (Brachyury T-box) [44]. Like the later specification of cardiogenic mesoderm, this earlier developmental choice point also appears to be regulated by a number of lncRNA.

Alexanian et al. looked at the regulation of three transcription factors during mesendoderm differentiation in mouse hanging drop embryoid body cultures: *Eomes, Gsc* and *Sox17* [45]. Each of these genes is regulated by an enhancer which produces an associated eRNA. CRISPR deletion of the *Eomes* enhancer/eRNA *Meteor* (mesendoderm transcriptional enhancer organizing region) resulted in loss of *Mesp1* expression together with a corresponding increase in the neural precursor gene *Neurg3* [45], indicating that cells were driven towards a neuro-ectodermal rather than a cardiogenic cell fate. Interestingly, while expression of these genes is low in pluripotent cells and is activated as they differentiate, the activity of the enhancer (as indicated by H3K27Ac) and expression of the eRNA showed the opposite relationship [45]. This suggests that the function of the enhancer/eRNA is to prime the locus, resulting in competence (to differentiate to mesendoderm) at the pluripotent stage.

Expression of *GSC* is linked to expression of the lncRNA *DIGIT* (divergent to GSC, induced by TGF-β family signalling; conserved in mice and humans) [46]. The coding and non-coding genes are transcribed from opposite and adjacent strands by a bi-directional promotor regulated by a distal enhancer bound by SMAD3 (and thus activated by NODAL/Activin signalling during mesendoderm induction). Loss of *DIGIT* expression leads to loss of endoderm gene expression and experiments showed that in this case it is expression of the lncRNA itself, and not the enhancer activity, that is required for *GSC* expression [46].

## 4. Patterning the Embryo: The NODAL Pathway

### 4.1. The NODAL Pathway Regulates Left-Right Patterning of the Cardiogenic Mesoderm

**Figure 2:**
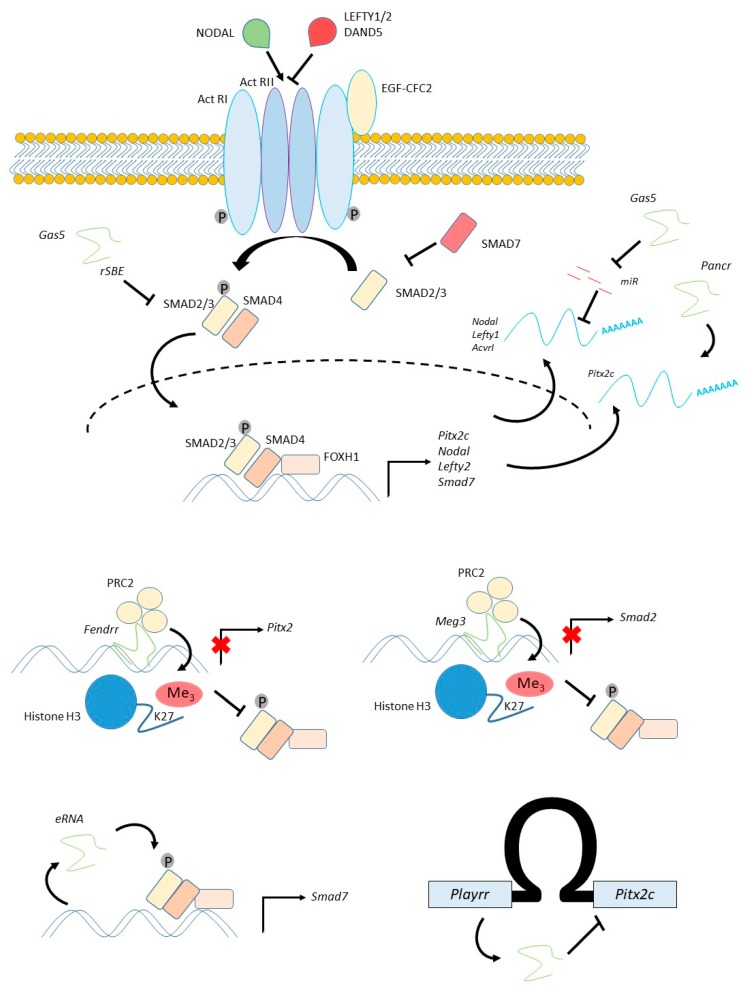
Long non-coding RNA acting within the NODAL signalling pathway. The TGFβ family protein NODAL (green) binds to a transmembrane receptor (ActRI, ActRII). Binding requires the co-factor EGF-CFC2 and can be inhibited by LEFTY1/2 or DAND5 (red). Receptor activation leads to phosphorylation of SMAD2/3, which recruits SMAD4 and translocates across the nuclear membrane (dashed line). SMAD2/3 can be inhibited by SMAD7. The SMAD2/3/4 complex binds DNA in cooperation with tissue-specific transcription factors such as FOXH1, leading to transcription of target genes including *Pitx2c, Nodal, Lefty2* and *Smad7*. mRNA are indicated in the diagram in blue, while long non-coding RNA (lncRNA) are indicated in green and miRNA in red. Cytoplasmic lncRNA known to regulate NODAL pathway genes include *Gas5* (growth arrest specific), which can bind to and inhibit SMADs through its RNA SMAD binding element (rSBE) or act as an endogenous competitor to inhibit miRNAs targeting *Nodal, Lefty2* or *Acvr1*. Nuclear lncRNA are illustrated in the lower part of the diagram. *Fendrr* (fetal-lethal noncoding developmental regulatory RNA) can recruit PRC2 (polycomb repressive complex 2) to the *Pitx2* locus, leading to trimethylation of histone H3 lysine 27. This inhibits binding by SMAD2/3/4 and prevents transcription. *Meg3* (maternal expressed gene) similarly recruits PRC2 to the *Smad2* locus. *Smad7* expression is regulated by an enhancer RNA (eRNA) which promotes expression. *Playrr* (Pitx2 locus-asymmetric regulated RNA) mediates a chromatin-looping mechanism to regulate *Pitx2* expression.

Following specification of the cardiac lineages, they must be given positional information and organised to form the tissues of the cardiovascular system. As an example of this process, we will here discuss patterning of left-right identity in cardiogenic precursors within the lateral plate mesoderm (LPM). This patterning process is critical for many aspects of subsequent cardiovascular morphogenesis, including asymmetric morphogenesis of the atria and vasculature, and its disruption is one of the causes of CHD [8,47,48]. The same pathway patterns precursors of the lung, resulting in its asymmetric development, and of the dorsal mesentery, asymmetric growth of which regulates looping of the gut. The NODAL signalling pathway, a member of the TGFβ family, determines left-right patterning (Figure 2). In the mouse embryo, *Nodal* becomes asymmetrically expressed on the left side of a midline organiser, called the node, in response to a cilia-generated fluid current flowing across the node, while an inhibitor, *Dand5* (also known as *Cerl2*) is asymmetrically expressed on the right side [49]. NODAL protein is then transferred to the left LPM where it activates expression of the *bicoid* related homeodomain transcription factor *Pitx2c,* as well as an inhibitor, *Lefty2*. NODAL binds to the Type II Activin receptor—binding requires the co-factor EGF-CFC2 (also known as Cryptic), and this leads to recruitment and phosphorylation of Type I Activin receptors [50,51]. Activation of the Type I receptor leads to phosphorylation of the receptor SMADs (SMADs 2 and 3), which heterodimerise with SMAD4 and translocate to the nucleus [50,51]. The activated SMAD complex binds to DNA with low affinity and low specificity [51]. Association of the complex with tissue-specific transcription factors, normally FOXH1 in the case of NODAL signalling, confers high-affinity binding and specificity [51,52]. The activated SMAD complex interacts with many chromatin remodelling complexes [53,54] and, in particular, can recruit the demethylase JMJD3 to counteract PRC2-mediated transcriptional repression at NODAL transcriptional targets as part of a complex regulatory feedback circuit leading to selective activation [55,56]. A number of protein-level feedback and control mechanisms also exist in the pathway, including aforementioned inhibitory TGFβ ligands, which competitively bind to and block the receptor (e.g., LEFTY1/2 [57]; DAND5 [49]) and inhibitory SMADs (e.g., SMAD7 [58]).

### 4.2. Regulation of Pitx2c

The expression of *Pitx2c* in cardiogenic precursors within the left LPM represents the endpoint of the NODAL pathway. *Pitx2c* expression is established in the left second heart field and then maintained here by NKX2.5 [59] (a component of the CGRN). *Pitx2c* expression is necessary for left-sided morphogenesis [60]. Whereas the expression of *Nodal* and *Lefty2* in the LPM is transient, *Pitx2c* expression persists into the adult in derivatives of the left LPM, including the left atrium.

**Figure 3:**
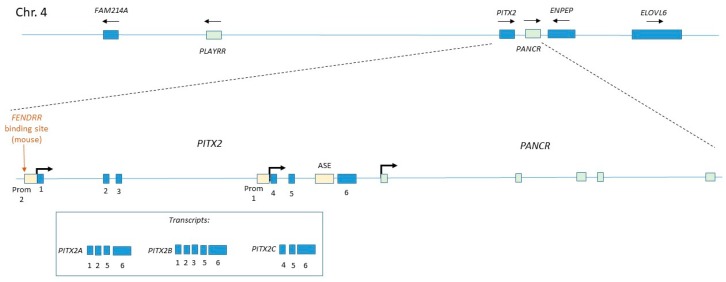
Organisation of the *PITX2/PANCR* (*PITX2* adjacent noncoding RNA)*/PLAYRR* (*PITX2* locus-asymmetric regulated RNA) locus. Blue boxes indicate protein-coding exons/genes, green boxes indicate transcribed non-coding loci and yellow boxes indicate regulatory regions of DNA. The location of the mouse *Fendrr* binding site is indicated. The lower box illustrates the three transcripts produced from the *PITX2* locus.

*Pitx2c* is transcribed from the *PITX2* locus, which has a complex organisation, having two promotors which initiate transcription beginning at different exons and producing different splice variants [61] (Figure 3). Left-sided *Pitx2c* expression is regulated by the asymmetric enhancer (ASE), which contains binding sites for FOXH1. The *Pitx2c* isoform regulates left-sided identity, while other isoforms are expressed symmetrically during development, largely in other organs but also at low levels in the heart [62].

A large number of lncRNA have been shown to regulate *Pitx2c/PITX2C* expression (Figure 2 and Figure 3). *PANCR* (*PITX2* adjacent noncoding RNA, also known as RP11–380D23.2) is located adjacent to the distal end of the human *PITX2* gene and is transcribed from the same strand [63] (Figure 3). *PANCR* genomic sequence is only partly conserved in mice, in which model there is no evidence for expression of a homologous lncRNA. *PANCR* and *PITX2* are co-ordinately expressed during both cardiogenic differentiation [63] and lung epithelial differentiation [64] of human embryonic stem cells (hESCs). During cardiogenic differentiation, both are expressed at a timepoint before the expression of the appearance of markers of differentiated cardiomyocytes [63], consistent with the known expression of mouse *Pitx2c* in cardiogenic precursors. In adults, expression is restricted to the left atrium (and the eye) [63]. *PANCR* short interfering RNA (siRNA) knockdown in hESC-derived cardiomyocytes also reduces *PITX2C* expression, indicating that *PANCR* is required for *PITX2C* expression [63].

Cell fractionation analysis of differentiated cardiomyocytes indicated that >95% of *PANCR* RNA was located in the cytoplasm [63], suggesting it may regulate expression at the post-transcriptional level. The *PANCR* locus contains a DNA motif which is bound by PARP1 (poly(ADP-ribose) polymerase 1) [64]. PARP1 is a histone-binding protein which modifies chromatin condensation [65]. Activated PARP1 leads to euchromatin formation. In differentiation assays towards lung epithelium, it was found that PARP1 is strongly expressed at the definitive endoderm stage, but downregulated in differentiated cells at the time that both *PITX2* (using primers that detect all isoforms) and *PANCR* are upregulated, and knockdown of *PARP1* upregulates both *PITX2* and *PANCR* expression [64]. Thus a model emerges, whereby PARP1 binding to the *PANCR* locus promotes chromatin condensation, inhibiting expression. When PARP1 expression is lost at later stages, this induces *PANCR* expression, which in turn acts in *cis* to regulate expression of the nearby protein-coding gene, perhaps post-transcriptionally.

Further cis regulatory mechanisms were uncovered in a study of the dorsal mesentery (a non-cardiac derivative of the LPM), in which system Welsh et al. uncovered an lncRNA-mediated programme of right-sided gene expression which negatively regulates *PITX2C* [66]. This work revealed that the protein-coding genes glutamyl aminopeptidase A (*ENPEP*), fatty acid elongase (*ELOVL6*) and the uncharacterised gene *FAM241A*, which are located adjacent to *PITX2* on chromosome 4 (in humans), are all expressed in the right LPM [66]. A lncRNA named *PLAYRR* (Pitx2 locus-asymmetric regulated RNA) is located at a distance of several hundred kilobases from *PITX2* within a gene desert region. *PITX2C* and *PLAYRR* exhibit mutual antagonism and loss of either gene results in bilateral expression of the other [66]. The *PLAYRR* promoter e926 contains *PITX2* binding sites. Welsh et al. demonstrated that these genes are part of a topologically-associated domain (TAD), or a self-interacting region of chromatin. Within this TAD, conformational changes which alter the distance between the *PITX2C* and *PLAYRR* loci regulate their expression, and these interactions require the sequence-specific scaffold protein, CCTF. Together, these data indicate a *cis*-regulatory mechanism is operating.

The lncRNA *Fendrr* (fetal-lethal noncoding developmental regulatory RNA) is bilaterally expressed in the LPM and in earlier primitive streak stage cardiogenic mesoderm [67]. *Fendrr* is located adjacent to the protein-coding gene *Foxf1* and transcribed on the opposite strand, with which gene it is co-expressed in LPM [67]. Loss of function mutants are embryonic or peri-natal lethal, and exhibit defects in the LPM and its derivatives such as the lung [67,68]. A four-chambered heart does form [67], although some new-borns have a ventricular septal defect [68]. No clear left-right patterning defects have been identified. Mutants express increased levels of CGRN genes such as *Nkx2.5* and *Gata6*, and this is associated with increased H3K4me3 at these loci (indicating open chromatin). Expression of *Pitx2* was increased by E9.5 in mutants, and showed a reduction in the repressive histone mark H3K27me3 as well as reduced occupancy of the PRC2 component EZH2 [67]. Grote et al. subsequently showed that *Fendrr* can bind to both the PRC2 and TrxG/MLL complexes [67]. PRC2 binding seems to be mediated by a 56-mer motif, which is structurally similar to an 89-mer PRC2-binding motif found in the previously mentioned *HOTAIR* lncRNA [69] and a 66-mer motif located in *Chaer* (cardiac-hypertrophy-associated epigenetic regulator), an lncRNA expressed in mouse cardiomyocytes [70].

The *Pitx2* promoter 2 (Figure 3) contains a region homologous to a second motif within *Fendrr*, and Grote et al. demonstrate that this mediates targeting to the genome through formation of a triplex between the single-stranded lncRNA and double-stranded DNA [67]. A similar *Fendrr*-binding motif exists within the promoter of *Foxf1* [67]. Thus, it seems that *Fendrr* regulates gene expression by recruiting histone modifying complexes to specific sites within target gene promoters.

Expression of *Fendrr* is transient, being largely downregulated by E9.5 [67]. Thus, it may function in left-right patterning to limit the expression of *Pitx2c* in the early embryo (through recruitment of PRC2), and its downregulation may serve as a release to permit later high-level *Pitx2c* expression. Alternatively, given that *Fendrr* binds to Promoter 2, which controls the expression of *Pitx2b* and *Pitx2a* (Figure 3), it may play a role in isoform-specific expression within the LPM by preventing transcription from this promoter. Finally, it is possible that *Fendrr* fine-tunes the expression level of *Pitx2c*; this is important because it has been shown that *Pitx2c* acts in a dose-dependent manner during the development of LPM-derivatives [71]. What is the function of FOXF1? FOXF1 is bilaterally expressed in the LPM, with mouse mutants exhibiting lung defects [72], but no obvious left-right patterning defect has been reported. However, given that the related factor FOXH1 co-operates with SMAD2/3/4 to activate genes downstream of *Nodal*, a similar SMAD-binding role for FOXF1 in this pathway cannot be ruled out.

### 4.3. Regulation of NODAL and SMAD Signalling

Although we know most about lncRNA regulation of *Pitx2c*, there is evidence that lncRNAs also act at other levels within the NODAL signalling pathway, and these suggest alternative mechanisms for lncRNA function (Figure 2).

The lncRNA *GAS5* (growth arrest specific) is highly expressed in pluripotent hESCs, where it is important in maintaining pluripotency by promoting NODAL signalling [73]. Knockdown of *GAS5* reduces *NODAL* expression, as well as several other components of the NODAL pathway, including its receptor *ACVRI* and the inhibitor *LEFTY1*, while *GAS5* over-expression has the opposite effect [73]. GAS5 appears to act as a competing endogenous RNA which binds to negative regulators of *NODAL* expression such as miR-2467, miR-3200 and miR-Let7e, functioning as a sponge to reduce their concentration and thus promote *NODAL* expression.

*GAS5* can also bind to proteins of the TGFβ/NODAL pathway, and in doing so, also appears to act as an endogenous competitor. RNA immunoprecipitation assays in mesenchymal stem cells demonstrated an interaction between *GAS5* and SMAD3 or SMAD4 [74]. *GAS5* contains multiple RNA SMAD-binding elements (rSBEs) which bind the SMAD3 protein and prevent it from binding to the promoter of *SM22* during vascular smooth muscle differentiation of mesenchymal stem cells [74]. Interestingly, these data also suggest that SMAD3 translocation to the nucleus is associated with nuclear export of *GAS5*. *GAS5* has also been shown to bind to another protein, glucocorticoid receptor, in a similar manner [75].

The lncRNA *MEG3* (maternal expressed gene), like *Bvht* and *Fendrr*, recruits PRC2 to target gene loci. In the breast cancer line [24,40], *MEG3* synchronously negatively regulates several TGFβ genes including *SMAD2* [76]. Like *Fendrr, MEG3* also targets regulatory regions via a short motif which forms triplex RNA–DNA [76]. These regions tend to be rich in GA nucleotides, and the RNA does not appear to form Watson–Crick base-pairs with the DNA.

While mammals have only a single *Nodal* gene, which has multiple functions during development, fish have multiple *Nodal* homologues, each performing a different function. One of these, *Squint*, is involved in mesendoderm induction and patterning, as in mammals, but also functions during dorsal patterning of the early embryo. This latter function has been shown to be mediated by the 3′ UTR of the mRNA independent of its protein-coding function [77]. The mechanism is unclear, although an endogenous competitor function has been ruled out [77]. Thus, *Squint* is an example of a bifunctional coding-noncoding gene [78]. A recent study of CRISPR/CAS9-mediated loss-of-function alleles in zebrafish failed to replicate the original findings [79]. 

DAND5 (also known as CERL2) is an inhibitory TGFβ family ligand expressed on the right side of the node during the earliest phase of left-right patterning and is critical for establishing left-sided *Nodal* expression. The asymmetric expression of *Dand5* mRNA occurs via a post-transcriptional mechanism and is dependent on an intact 3′ UTR [80]. This decay is mediated by a feedback regulatory loop involving WNT signalling [80], however, the precise molecular mechanism of mRNA degradation has not been determined. Given what we now know about lncRNA, it seems quite possible that lncRNA will be shown to play a role in this process.

SMAD7 is an inhibitory SMAD which blocks the interaction of SMAD2 with the activated Type I receptor [58]. *Smad7* is also transcriptionally activated by SMAD2/4 downstream of NODAL signalling [81], as are other inhibitors of the pathway, such as *Lefty2*, indicating that it functions in a regulatory feedback circuit. The *SMAD7* gene is regulated by an enhancer-associated eRNA located adjacent to the coding gene, and GapmeR-mediated knockdown of this lncRNA in neonatal cardiac fibroblasts results in reduced *SMAD7* expression [39].

In summary, there is evidence that NODAL signalling can be regulated at multiple levels by lncRNA, but as yet there is no direct evidence for a role in left-right patterning of the cardiogenic mesoderm.

## 5. lncRNA and Congenital Heart Disease: Challenges and Prospects

### 5.1. Lack of Evidence Pointing Towards A Role in CHD

In Section 3 and Section 4 above, we have demonstrated that lncRNA are present at all stages of embryonic heart development and have described how they play central roles in many developmental processes. It is therefore somewhat surprising that very few lncRNA have been definitively proven to play a role in CHD. Of the lncRNA discussed above, the best candidates for a disease role are perhaps those regulating the left-determining gene *PITX2* (discussed in Section 4.2 above). This is because atrial fibrillation risk has been shown to be correlated with four single nucleotide polymorphism (SNPs) located close to *PITX2* [63]. The SNPs do not map to any known lncRNA and do not correlate with *PANCR* expression in adult left atrium tissue [63], but are located within a noncoding intergenic region.

There may be several reasons for this lack of evidence. One is that genome-wide screens have until recently focused on the exome and have not sequenced intronic or intergenic regions. Another reason is that several features of lncRNA render them impervious to interrogation by standard methods, making progress in this field slow. These include their lack of cross-species sequence conservation, general low level of expression, and in some cases their absence in the cytoplasm.

### 5.2. Challenges in lncRNA Research

#### 5.2.1. The Problem of Low Cross-Species Sequence Conservation

lncRNA genes are less conserved than protein-coding genes and exhibit a high level of turnover, indicating rapid evolution. For example, a comparison of rodents demonstrated that approximately half of intergenic lncRNA have been gained or lost since mice and rats diverged from a common ancestor [82]. A comparative study of 11 tetrapod species revealed that over 80% of human-transcribed lncRNA are primate-specific, only 19% are found in mice and 3% in *Xenopus* [83]. Furthermore, transcriptional turnover is also higher for lncRNA than for coding genes, that is to say that in some cases syntenic sequence can be identified in two genomes but the corresponding RNA is expressed in only one species [82]. This appears to be the case for *Bvht*, as syntenic sequence can be seen in the human genome, but no evidence for transcription has been found.

This lack of sequence conservation presents problems to the developmental biologist hoping to determine the function of a given lncRNA using classical loss-of-function analysis in model organisms. The situation is further complicated by the fact that many lncRNA overlap coding sequences or are expressed from their enhancers, so that even in cases where a conserved gene does exist it may not be possible to make a specific targeted deletion. The use of CRISPR gene editing is useful to target mutations to specific genomic positions, for example, to the transcriptional start site, in order to limit disruption of adjacent genes. Furthermore, emerging CRISPR technology allows targeting of RNA rather than DNA to generate loss-of-function mutations [84].

For these reasons, *in vitro* models are often used to study lncRNA. However, *in vitro* models are limited in their ability to model complex processes of morphogenesis and patterning, as well as technical limitations related to gene knockdown discussed below.

#### 5.2.2. The Problem of Targeting Nuclear RNA Expression

Many of the *in vitro* studies described herein utilise transfection of either short hairpin RNA (shRNA) or synthetic short interfering RNA (siRNA) to knock-down gene expression. Transfection of siRNA often has only a transient effect, especially in dividing cells such as ESCs. To achieve a more stable knockdown, a viral or plasmid-based vector may be used to drive shRNA expression. The resulting RNA is transcribed by RNA polymerase III to produce a stem-loop structure resembling a pri-miR, which is then processed by DROSHA and DICER and exported from the nucleus utilising the cell endogenous miR pathway. Gene silencing requires loading into the RISC complex together with AGO2. Because the RISC complex is located in the cytoplasm, gene silencing is most effective for cytoplasmic RNA. A number of studies have demonstrated low efficiency of siRNA knockdown of nuclear RNA, such as small nucleolar RNA (snoRNA) [85]

Components of the RISC complex including AGO2, Dicer and GW182 have been demonstrated within the nucleus of human HeLa cells [86]. This work showed that nuclear AGO2 is bound to miR, with 75% of cytoplasmic miRs also being found in the nucleus, and furthermore that siRNA targeting the nuclear lncRNAs *MALAT1* and *NEAT1* effectively knocks down nuclear expression [86]. Nuclear eRNA [87,88] and small nuclear RNA [89] have also been effectively knocked down using siRNA transfection.

An alternative approach is to use a GapmeR antisense oligonucleotide. These synthetic constructs consist of a central portion of single-stranded antisense DNA oligonucleotide flanked by locked-nucleic acid sequences conferring protection from degradation [90]. Binding of the ssDNA to an RNA molecule triggers RNase H mediated degradation of the DNA–RNA heteroduplex. RNase H is normally present in the nucleus, where it is important for excision repair, and therefore GapmeR-based silencing does not require cytoplasmic proteins to enter the nucleus. These have been successfully used to knock down nuclear-localised lncRNA including eRNA [91,92]. GapmeR-mediated knockdown appears to be more efficient for nuclear knockdown than siRNA [92,93], although siRNA is more efficient for cytoplasmic lncRNA [93].

It is important that studies of lncRNA exhibiting a mixed nuclear/cytoplasmic distribution verify that expression is reduced in both cellular compartments. This is a criticism of some work described above, for example, Klattenhoff et al. [24] used shRNA to knockdown *Bvht* in ESCs, before describing a nuclear mechanism of action, but failed to verify nuclear knockdown.

#### 5.2.3. The Problem of Low Endogenous Expression of lncRNA

It is well known that many lncRNA are expressed at low levels, often an order of magnitude lower than coding RNA. As well as presenting difficulties in detection, this can also present difficulties for knockdown experiments. Because lncRNA are expressed at a low level, it is reasonable to assume that they can function at low levels. siRNA-knockdown is not 100% efficient, and residual expression will always remain, limiting the utility of these studies.

#### 5.2.4. The Problem of Identifying lncRNA Binding Partners

The identification of lncRNA binding partners generally relies on immunoprecipitation of either a predicted protein interactor or of an epitope-tagged expressed RNA. For example, Klattenhoff et al. identified the interaction between *Bvht* and PRC2 by expression biotin labelled RNA [24]. However, overexpression of an ectopic RNA can lead to false positives and *in vivo* immunoprecipitation, such as that used by Grote et al. to identify the interactions between *Fendrr* and PRC2/TrxG, is preferable [67].

Logically, one might predict that it is the secondary structure of an RNA that determines its binding specificity and therefore that if we were able to accurately predict this structure we might be able to predict function. There is some evidence that secondary structure may be better conserved between species than the primary sequence of an lncRNA. For example, the previously mentioned *Gas5* shows 70% sequence conservation between human and mouse, but strong conservation in the secondary structure of the glucocorticoid receptor binding domain [75]. Similarly, rat *Gas5* shows only 12% sequence similarity to the mouse, but retains the SMAD binding element [74].

## 6. Diagnostics Applications of lncRNA

A biomarker is a naturally occurring molecule, gene or characteristic that allows objective measurement of a normal biological process, a pathogenic process or pharmacologic responses to a therapeutic intervention. Much interest over the last few years has focused on the use of miRs as biomarkers, particularly in exosome-derived miR, as these extracellular vesicles can be extracted from blood and other body fluids and are actively secreted by most cells, thus potentially providing a sophisticated readout of health [94]. lncRNA have received less attention. One problem with lncRNA as a biomarker is that it is generally expressed at a very low level, making detection technically challenging. However lncRNA has been detected within exosomes produced, for example, from vascular endothelium [95] indicating their potential as a biomarker.

A number of studies have suggested circulating lncRNA (exosomal or non-exosomal) may be a useful biomarker for myocardial infarction and other adult heart diseases. For example, in a multicentre case-control study, Kumarswamy et al. found that circulating lncRNA levels are altered following myocardial infarction and furthermore that the mitochondrial lncRNA *LIPCAR* (long intergenic noncoding RNA predicting cardiac remodelling) has prognostic value as an indicator of future heart failure and patient mortality [96]. Another study suggested that plasma levels of *LIPCAR*, together with another lncRNA *H19*, may predict the risk of coronary artery disease [97].

In the cancer field, Exosome Diagnostics Inc. market a commercial diagnostic kit for prostate cancer based on the analysis of an exosome lncRNA, *PCA3* [98], indicating that exosome lncRNA biomarkers can have diagnostic value.

It is possible that some of the circulating lncRNA biomarkers identified for myocardial infarction are simply released into circulation following cell damage, and are therefore conceptually similar to measurement of cardiac troponin, rather than being actively released. CHD biomarkers are more complex, as any biomarker originating from the foetus would need to cross the placental barrier, and therefore is more likely to be part of an active signalling pathway rather than a by-product of cellular damage. Perhaps for this reason, studies of the predictive value of lncRNA as a biomarker for CHD are presently limited. However, in one study circulating lncRNA was extracted from the plasma of pregnant women at 24–25 weeks gestation and assessed by microarray analysis [99]. A test group of 62 women carried foetuses diagnosed via echochardiography with a CHD (about half of cases had a ventricular septal defect, followed in frequency by atrial septal defect and Tetralogy of Fallot) was compared to a control group of 62 women with a normal pregnancy. Over 3000 up- and down-regulated lncRNAs were found to show a >2 fold change. Five lncRNA changes were validated by qPCR, including *FAM230H* and *H19*. *H19* is one of the best characterised of all lncRNA—it regulates cell proliferation through the IGF2 pathway and is known to be involved in the response to vascular injury. One problem with *H19* as a biomarker is that it has been implicated in so many diseases that specificity may be limited.

An analysis of cardiac tissue extracted from aborted foetuses at 17–20 weeks of gestation with or without ventricular septal defect (VSD) identified 880 upregulated and 628 downregulated lncRNAs [100]. Song et al. narrowed down this list by selecting for conserved lncRNA located near protein-coding genes showing heart-specific expression. This resulted in the identification of two potential biomarkers: *ENST00000513542* is an antisense lncRNA transcribed from the *SMAD1* locus, while *RP11-473L15.2* is located close to *FGF10* [100]. SMAD1 is an effector of BMP signalling. FGF10 is a marker of the second heart field and BMP signalling is also important in SHF development [101], thus these data point towards a role for the SHF in VSD. A second study focused on expression of a single lncRNA, HA117, in right ventricle outflow tract tissue of patients with a mean age of 13 months undergoing surgery to correct Tetralogy of Fallot [102]. This study indicated that the expression level of HA117 is correlated with disease severity [102]. Whether these biomarkers are detectable in plasma was not determined in either study.

This question was partly addressed by another study which focussed on a single lncRNA (*HOTAIR*), but compared expression levels in heart tissue to circulating plasma levels [103]. Right atrial appendages were taken from 16 CHD patients requiring surgery with a mean age of 12. This was compared to the same tissue from 14 patients also requiring surgery, but for the inflammatory condition rheumatic valvular disease, and with a mean age of 46. In a parallel study, plasma was taken from 90 patients with CHD (including VSD, ASD and persistent ductus arteriosus) as well as 36 controls. The results showed both upregulation of *HOTAIR* in atrial appendages of congenital versus adult heart disease and in the plasma of patients with all forms of CHD versus controls. There were many problems with this study, which could call into question its validity. The two groups compared for the tissue analysis were of very different ages and not directly comparable. In addition, a single *HOTAIR* primer pair was used in these assays with no attempt at independent validation. This study is perhaps most remarkable, in common with the study by Wang et al. [102], because so much effort was made to obtain these valuable samples from which only one lncRNA assay was performed. It is unclear why the authors chose not to perform a global expression analysis.

Genome-wide association studies search for genetic variants associated with disease, which may be useful in predicting the risk of having a child affected by CHD. Many disease-associated single nucleotide polymorphisms have been found to map to non-coding regions of the genome, and it is possible that some of these may be found to map to lncRNAs. In a study of Han Chinese children, ranging in age from 1 month to 2.5 years and exhibiting a spectrum of CHD including VSD, ASD and ToF, a significant association was identified with a SNP located within the lncRNA *MALAT1* (metastasis-associated lung adenocarcinoma transcript 1) [104].

## 7. Therapeutics Applications of lncRNA

Given their key roles in cardiogenic precursor lineage specification, there is clearly therapeutic potential in the use of lncRNA to generate cardiac cells for regenerative medicine. Driving expression of *Bvht* has been shown to improve the efficiency of cardiogenic differentiation of mouse bone marrow-derived mesenchymal stem cells [105]. However, given that the existence of a human homologue of *Bvht* has been questioned [24], it is unclear how useful this will be for regenerative medicine. *Bvht* sequence is present in the human genome—we performed a BLAST search with the transcribed mouse sequence against the human genome and found evidence that the sequence is at least partially conserved, however, there is, as of yet, no evidence for human expression.

An example of the translational possibilities offered by targeted knockdown of lncRNA *in vivo* comes from a study of cardiac remodelling and heart failure. The lncRNA *CHAST* (cardiac hypertrophy–associated transcript) promotes hypertrophy and is upregulated both in a mouse model of hypertrophy and in hypertrophic cardiac tissue from aortic stenosis human patients [106]. *In vivo* GapmeR-based silencing of *CHAST* prevented pathological remodelling in the mouse model [106].

A number of studies have investigated the possibility of using lncRNA manipulation to promote cardiomyocyte proliferation as a way to repair the infarcted heart. Targeted knockdown of the lncRNA *SIRT1 antisense* using adeno-associated virus expression of locked nuclei acid antisense inhibitors was found to promote cardiomyocyte proliferation and reduce infarct size following myocardial infarction [107]. Viral overexpression of another lncRNA, *NR_045363* (1700024F13Rik), in 7-day-old mouse heart could improve cardiac function and stimulate cardiomyocyte proliferation after myocardial infarction [108]. This lncRNA appears to act as an endogenous competitor for miR216a and regulates JAK-STAT signalling [108]. Mice carrying a targeted deletion of the lncRNA *Cpr* (cardiomyocyte proliferation regulator) show increased cardiomyocyte proliferation, improved myocardial function and reduced scar formation following myocardial infarction [109].

Recently, evidence has emerged that miRNA is present in the nucleus [86,89], and furthermore that it might interact with eRNA in order to stimulate transcription by defined enhancers [110]. Therefore, using such nuclear miRs to target eRNA might in the future provide a possible novel route for therapeutics.

In summary, these studies illustrate the potential translational value of lncRNA manipulation, but therapeutic applications to CHD are currently lacking.

## 8. Conclusions and Perspectives

CHD is the most common birth defect and affects almost 1 in every 100 new-borns, yet our understanding of the genetic causes of CHD is still relatively poor and only a minority of cases can be ascribed to a clear genetic defect [111]. It is becoming increasingly clear that in the hunt for disease loci, we must consider not only the protein-coding genome but also the multiplicity of regulatory regions and the non-coding RNA associated with these loci. In this review, we have discussed how non-coding RNA provides important feedback control within many developmental processes, including cardiogenic lineage restriction and patterning of the emerging cardiogenic fields. The direct proof for a role for lncRNA in specific CHDs is still largely lacking, but the evidence coming from the study of model organisms and in vitro differentiation indicates that these molecules play critical roles, suggesting that it will be found. This current disparity may reflect the complexity of the regulatory circuits at play, as well as a prior focus on exome rather than whole-genome sequencing.

## Figures and Tables

**Table 1:** Summary of lncRNA discussed in the text.

lncRNA	Model System(s) Used	Expression Profile	Nuclear/Cytoplasmic	Loss of Function Methods	Loss of Function Phenotype	Target Genes	Protein Interactions	Evidence for lncRNA Expression in Humans	Reference
*Bvht*	Mouse (ESC/EB)	ESC, Mesoderm, Cardiogenic mesoderm, Adult heart	Both	shRNA	Loss of beating CMs in EB culture	*Mesp1*	PRC2 CNBP	RNA-seq from adult heart indicates lack of expression in human and rat, suggesting *Bvht* is mouse-specific [24]	[24,105]
*Carmen*	Mouse PC19CL6, Human foetal ventricle CPC	Cardiogenic mesoderm, Early heart tube, Adult heart, VSMC	Nuclear	shRNA		*Bvht* *Eomes* *Oct4* *Nanog*	PRC2	RNA-seq from adult heart indicates expression conserved in human and rat. Expressed in ventricle of week 12 foetus	[24,40]
*Linc1405*	Mouse (ESC/EB)	Cardiogenic mesoderm, Heart (E10.5, E16.5)	Nuclear	shRNA	Loss of beating CMs in EB culture	*Mesp1*	TrxG EOMES	ND	[42]
*Meteor*	Mouse (ESC/EB)	ESCs	Nuclear	CRISPR	Lack of mesoderm specification, Gain of ectodermal specification	*Eomes*	ND	ChIP-seq data suggests transcription is conserved	[45]
*Digit*	Mouse/Human (ESC/EB)	Endoderm	Nuclear	shRNA LNA CRISPR	Lack of endoderm	*Gsc*	ND	Expressed in hESC derived endoderm	[46]
*PANCR*	Human (ESC/EB)	Cardiogenic mesoderm, Lung epithelium, Adult left atrium, Adult eye	Cytoplasmic	siRNA	Loss of *Pitx2* expression	*Pitx2*	PARP1	Human-specific gene. No evidence of mouse homologue	[63,64]
*PLAYRR*	Mouse/chick (embryo)	Right-sided dorsal mesentery	Nuclear	CRISPR	Gain of *Pitx2* expression	*Pitx2*	CTCF	Conserved in human	[66]
*FENDRR*	Mouse (embryo)	Cardiogenic mesoderm, Lateral plate mesoderm (caudal, bilateral), Adult lung	Nuclear	Transgenic inducible shRNA	No phenotype	*Pitx2* *Foxf1*	PRC2 TrxG	Expression of human homologue confirmed in numerous studies	[67,68,112]
				Null allele 1	Embryonic lethal, Myocardial hypoplasia				
				Null allele 2	Perinatal lethality, VSD lung maturation and vascularization defect				
*GAS5*	Human (ESC)	ESC Mesenchymal stem cells	Both	shRNA	Differentiation of ESCs, Differentiation of VSMCs	TGFβ/Nodal pathway	SMAD3	Expressed in human ESCs	[73,74,75]
*MEG3*	Human (BT-549 cells)	Breast cancer	Nuclear	siRNA	Gain of TGFβ expression	TGFβ/Nodal pathway	PRC2	Expressed in human BT-549	[76]

Abbreviations: *Bvht* = Braveheart; *Carmen* = cardiac mesoderm enhancer-associated noncoding RNA; CM = cardiomyocyte; CNBP = CCHC-type zinc finger nucleic acid binding protein; CPC = cardiac progenitor cell; *Digit* = divergent to GSC, induced by TGF-β family signalling; EB = embryoid body; Eomes = Eomesodermin; ESC = embryonic stem cell; Gsc = Goosecoid; HR = homologous recombination; hESC = human embryonic stem cells; LNA = locked nucleic acid; *Meteor* = mesendoderm transcriptional enhancer organizing region; *MEG3* = maternal expressed gene; ND = not determined/no data; *PANCR* = *PITX2* adjacent noncoding RNA; PARP1 = poly(ADP-ribose) polymerase 1; PRC2 = polycomb repressor complex 2; siRNA = short interfering RNA; shRNA = short hairpin RNA; TrxG = trithorax group chromatin modifying complex; VSD = ventricular septal defect; VSMC = vascular smooth muscle cell.
